# Pain, disability and impairment associated with podiatric problems in patients with acute gout

**DOI:** 10.1186/1757-1146-4-S1-P21

**Published:** 2011-05-20

**Authors:** Mike Frecklington, Keith Rome, Peter McNair, Nicola Dalbeth

**Affiliations:** 1 Health and Rehabilitation Research Centre, Faculty of Rehabilitation and Occupational Studies, School of Podiatry, Auckland University of Technology, Auckland 0627, New Zealand; 2 Health and Rehabilitation Research Centre, Faculty of Rehabilitation and Occupational Studies, School of Physiotherapy, Auckland University of Technology, Auckland 0627, New Zealand; 3 Department of Medicine, Faculty of Medical and Health Sciences, University of Auckland, Auckland 1023, New Zealand

## Background

Gout is on the increase worldwide and is the most common form of inflammatory arthritis affecting men. Gout is of particular significance to New Zealand, affecting approximately 15% of Maori and Pacific men in South Auckland, thus it has been dubbed the ‘gout capital of the world’. Gout most frequently affects the foot, with initial attack affecting in the first metatarsophalangeal joint (1^st^ MPJ) in 50% of patients. Involvement of the first 1^st^ MPJ eventually occurs in 90% of individuals with gout. Despite the predilection of gout to the foot, the impact of gout on foot function is not well understood. A recent study investigating patients with chronic gout found the foot to be a rigid, high-arched with limited range of motion at the major joints of the foot. An increased threshold of pain, disability and a decrease in function was also reported. These changes associated with chronic gout may contribute to further progression of pain, disability and impairment. The impact of acute gout on musculoskeletal function has not been examined in a detailed or objective manner. The aim of the current study is to assess the impact of acute gout on foot pain, function/impairment and disability.

## Methods

An observational follow-up design is currently being used to assess the impact of acute gout, with the first study visit occurring when a patient is having an acute flare and the second study occurring during the intermittent/chronic period. Patients are recruited from both primary and secondary care settings. Patient specific outcome measures assess pain, function/impairment and disability. Data obtained from initial visit and 6 weeks after first flare sits will help to determine the impact of acute gout and also make comparisons between acute and chronic gout.

## Results

Data collection to date has captured 13 patients (mean age: 50.3 years, mean gout duration: 13.3 years) during an acute flare. A further 5 patients have declined to be included into the study. Findings so far indicate that acute gout flares are characterised by increased pain, disability and impairment compared to periods of intermittent gout. Difficulties in footwear selection are a recurring theme with acute gout patients. Problems with footwear selection appear to be related to foot dimensions exceeding those of footwear available to this patient group.

## Conclusions

In terms of podiatric intervention and management plans for gout patients, it appears that little can be done during acute flares due to excruciating levels of pain apart from pharmacological interventions. However, interventions relating to footwear and improving foot function may be beneficial during the stages of intermittent/chronic gout. The presentation will focus on issues of patient recruitment in culturally sensitive groups and will describe the current trends of pain, disability, function and impairment.

